# Immunohistochemical Pattern of Histone H2A Variant Expression in an Experimental Model of Ischemia–Reperfusion-Induced Acute Kidney Injury

**DOI:** 10.3390/ijms24098085

**Published:** 2023-04-30

**Authors:** Jelena Nesovic Ostojic, Maja Zivotic, Sanjin Kovacevic, Milan Ivanov, Predrag Brkic, Nevena Mihailovic-Stanojevic, Danijela Karanovic, Una Jovana Vajic, Zoran Miloradovic, Djurdjica Jovovic, Sanja Radojevic Skodric

**Affiliations:** 1 Department of Pathological Physiology, Faculty of Medicine, University of Belgrade, 11000 Belgrade, Serbia; sanjin.kovacevic@med.bg.ac.rs; 2 Institute of Pathology, Faculty of Medicine, University of Belgrade, 11000 Belgrade, Serbia; maja.zivotic@med.bg.ac.rs; 3 Institute for Medical Research, Department of Cardiovascular Physiology, National Institute of Republic of Serbia, University of Belgrade, 11000 Belgrade, Serbia; ivmilan@imi.bg.ac.rs (M.I.); nevena@imi.bg.ac.rs (N.M.-S.); danijela.karanovic@imi.bg.ac.rs (D.K.); unajovana@imi.bg.ac.rs (U.J.V.); zokim@imi.bg.ac.rs (Z.M.); djurdjica@imi.bg.ac.rs (D.J.); 4 Department of Medical Physiology, Faculty of Medicine, University of Belgrade, 11000 Belgrade, Serbia; predrag.brkic@med.bg.ac.rs

**Keywords:** AKI, ischemic–reperfusion injury, histone, γH2AX, H2AZ, immunohistochemistry

## Abstract

Ischemia–reperfusion injury (IRI) is a frequent cause of AKI, resulting in vasoconstriction, cellular dysfunction, inflammation and the induction of oxidative stress. DNA damage, including physical DNA strand breaks, is also a potential consequence of renal IRI. The histone H2A variants, primary H2AX and H2AZ participate in DNA damage response pathways to promote genome stability. The aim of this study was to evaluate the immunohistochemical pattern of histone H2A variants’ (H2AX, γH2AX(S139), H2AXY142ph and H2AZ) expression in an experimental model of ischemia–reperfusion-induced acute kidney injury in spontaneously hypertensive rats. Comparing the immunohistochemical nuclear expression of γH2AX(S139) and H2AXY142ph in AKI, we observed that there is an inverse ratio of these two histone H2AX variants. If we follow different regions from the subcapsular structures to the medulla, there is an increasing extent gradient in the nuclear expression of H2AXY142ph, accompanied by a decreasing nuclear expression of γH2AX. In addition, we observed that different structures dominated when γH2AX and H2AXY142ph expression levels were compared. γH2AX was expressed only in the proximal tubule, with the exception of when they were dilated. In the medulla, H2AXY142ph is predominantly expressed in the loop of Henle and the collecting ducts. Our results show moderate sporadic nuclear H2AZ expression mainly in the cells of the distal tubules and the collecting ducts that were surrounded by dilated tubules with PAS (periodic acid–Schiff stain)-positive casts. These findings may indicate the degree of DNA damage, followed by postischemic AKI, with potential clinical and prognostic implications regarding this condition.

## 1. Introduction

Acute kidney injury (AKI) is a global health problem defined as a sudden decrease in kidney function and affects up to two-thirds of patients in intensive care units [1,2]. This clinical syndrome is accompanied by poor prognosis, prolonged hospitalization time, increased risk of progression to chronic kidney disease and a possible fatal outcome [3,4,5]. There is a strong connection between hypertension and AKI because increased blood pressure is a risk factor for acute kidney injury, but it is also a common comorbidity in patients with AKI [6]. Ischemia–reperfusion injury (IRI) is one of the most common causes of AKI, resulting in vasoconstriction, endothelial dysfunction, inflammation and the induction of oxidative stress [7,8]. DNA damage, including oxidative base modifications and physical DNA strand breaks (DSBs), is also a potential consequence of renal IRI [9].

Histones are highly conserved proteins that are important for packaging DNA into chromatin, which has nucleosomes as its monomeric subunits [10,11]. The nucleosome core particle consists of 147 bp of DNA wrapped around the histone octamer consisting of two of each of the core histones H2A, H2B, H3 and H4 [12]. Chromatin decreases the accessibility of DNA and consequently interferes with many biological processes, such as transcription, replication and repair, but helps to protect the DNA from damage by different kinds of stress [13]. Chromatin is subject to a posttranslational modification of the histone proteins, including acetylation, methylation, phosphorylation, ubiquitylation, glycosylation, ADP-ribosylation and carbonylation, and plays a major role in regulating gene expression [14]. The histone H2A variants, primary H2AX and H2AZ participate in DNA damage response (DDR) pathways to promote genome stability during exposure to different endogenous and exogenous harmful factors [10]. When DNA is damaged, DSBs are formed, and this is followed by the phosphorylation of the histone H2AX at serine 139. This modified form is termed gamma H2AX, (γH2AX) [15,16,17]. Because of the sensitivity and utility of γH2AX for the detection of DNA double-strand breaks, γH2AX has been identified as a potentially useful biomarker with clinical implications [18] and as a therapeutic marker for improving the efficiency of radiation, drug and other therapies [19,20,21].

In higher eukaryotes, the Williams–Beuren syndrome transcription factor kinase (WSTF) constitutively phosphorylates H2AX at its C-terminal residue, tyrosine 142; this phosphorylated H2AX is termed H2AXY142ph (H2AXY142) [22]. The H2AXY142ph modification is reversed after DSBs by EYA phosphatase-induced dephosphorylation. Disruption of any of these events affects the DNA damage response, leading to increased cell death following DNA damage [22,23,24,25]. Nucleosomes containing the histone variant H2AZ are important for gene transcription initiation and termination, chromosome segregation and DNA double-strand break repair, among other functions [26]. The histone variant H2AZ is also a master regulator of epithelial–mesenchymal transition [27].

Ischemia–reperfusion-induced acute kidney injury is characterized by DSBs. On the other hand, IR-induced acute kidney injury is a potentially reversible condition but carries increased risk of progression to chronic kidney disease that leads to fibrosis and decreased renal function. On the other hand, γH2AX might be useful in cancer therapy, for the evaluation of cells’ radiosensitivity, as an indicator of environmental health risks, elucidating the pharmacodynamics of cytotoxic drugs [17], but it was not investigated in AKI.

Considering all that was said above, the aim of this study was to evaluate the immunohistochemical pattern of histone H2A variant (H2AX, γH2AX, H2AXY142ph and H2AZ) expression in an experimental model of ischemia–reperfusion-induced acute kidney injury in spontaneously hypertensive rats (SHRs).

## 2. Results

### 2.1. Determining the Estimated Glomerular Filtaration Rate (eGFR)

**Figure 1:**
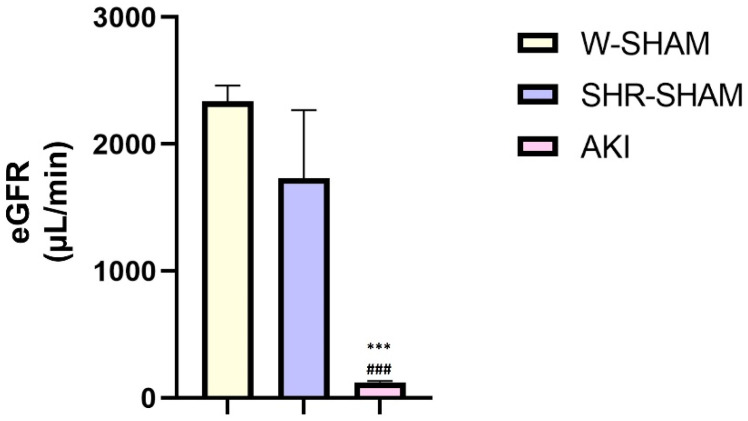
Estimated glomerular filtration rate in different experimental groups. One-way ANOVA with Tukey’s HSD post hoc test, ***, ^###^ *p* < 0.001.

In the AKI group, a significantly reduced eGFR was noticed in comparison to the sham-operated group (*p* < 0.001, Figure 1), confirming that ischemia–reperfusion injury was induced in the experimental rats.

### 2.2. Immunohistochemical Nuclear Expression of Histone γH2AX(S139) in SHR Kidneys and during AKI

Epigenetic histone modifications with regard to the presence of histone γH2AX(S139) were detected both in the sham-operated SHRs (control group) and in the rats with induced AKI. However, the extent of expression and some distribution patterns were different.

**Figure 2:**
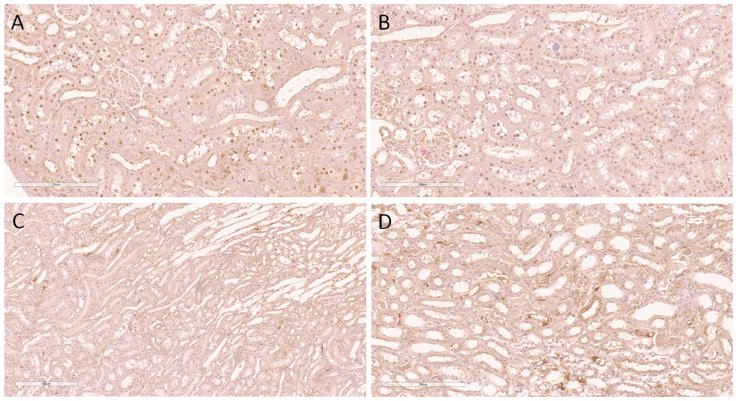
Immunohistochemical nuclear expression of histone γH2AX(S139) in sham-operated SHRs. (**A**) Expression in subcapsular kidney cortex involving the majority of nuclei of proximal tubulocytes; (**B**) expression in middle cortical zone detected in the same structures but with slightly decreased abundance; (**C**) juxtamedullary cortical zone with nuclear positivity in less than 25% of tubulocytes; (**D**) absence of expression in the kidney medulla. Magnification ×200 in all slides.

Figure 2 illustrates the expression of γH2AX(S139) in the sham group. It was noticed that this histone was expressed exclusively in the nuclei of proximal tubular epithelial cells, with varying extent through the cortical kidney parenchyma. Thus, the expression was diffusely detected within the subcapsular kidney cortical area, involving the majority of the nuclei of proximal tubulocytes (Figure 2A). In the middle cortical zone, expression was also detected in the same structures but with slightly decreased abundance (Figure 2B). Furthermore, the juxtamedullary cortical zone was also positive for γH2AX(S139) but mostly involved less than 25% of tubulocytes in the zone (Figure 2C). Since the medulla does not contain proximal tubules, this kidney zone was completely negative with regard to γH2AX(S139) expression (Figure 2D).

Considering that the expression of γH2AX(S139) was observed in sham-operated SHRs (control group), we also intended to explore the presence of this histone in sham-operated, normotensive Wistar rats (Figure S1). Here, we found a distribution pattern similar to that described in sham-operated SHRs (Figure 2).

**Figure 3:**
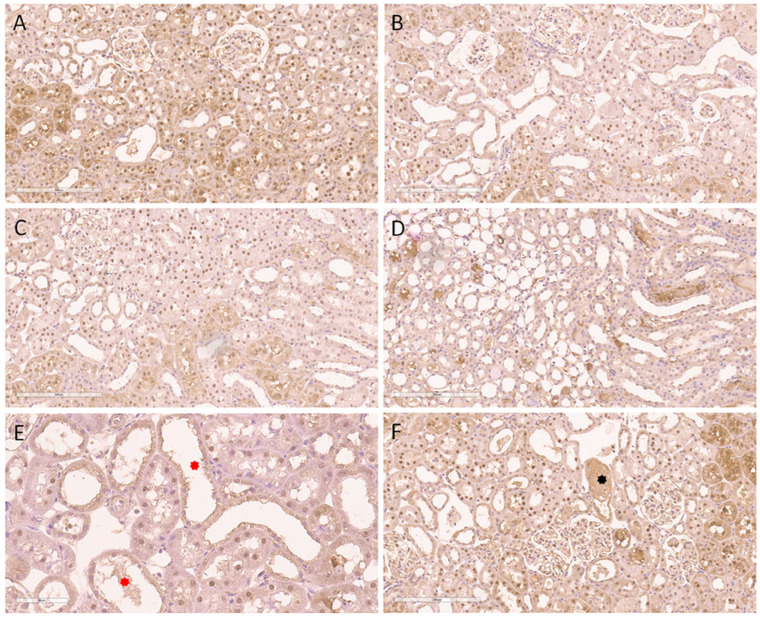
Immunohistochemical nuclear expression of histone γH2AX(S139) in animal AKI model. (**A**) Diffuse expression in subcapsular kidney cortex involving the majority of nuclei of proximal tubulocytes; (**B**) abundant expression in middle cortical zone detected in the same structure; (**C**) juxtamedullary cortical zone with nuclear positivity in up to 50% of tubulocytes; (**D**) absence of expression in the kidney medulla; (**E**) absence of expression in dilated proximal tubules, with flattened epithelium (red asterisks), as an early morphological sign of AKI; (**F**) absence of nuclear expression in the most severely affected tubules, such as those with necrosis of tubulocytes (black asterisk). Magnification ×200 (**A**–**D**,**F**), ×400 (**E**).

A significantly higher extent of expression was detected in the nuclei of proximal tubulocytes through the cortex within the model of AKI. Thus, Figure 3 shows the expression pattern through the cortical region and in the kidney medulla of the aforementioned animals. Compared to the sham group, the expression was more pronounced in the subcapsular region (Figure 3A) and evidently more diffuse in the middle cortex (Figure 3B), as well as in the juxtamedullary cortical zone (Figure 3C). Indeed, the kidney medulla was also devoid of γH2AX(S139) expression (Figure 3D). Despite the expression of γH2AX(S139) in the same structures (nuclei of proximal tubular epithelial cells), AKI induced a more widespread appearance through the whole kidney cortex. Nevertheless, the dilatation of proximal tubules, with a flattened epithelium, as a morphological sign of AKI, was observed without the expression of γH2AX(S139) in the nuclei (Figure 3E). The absence of nuclear γH2AX(S139) expression was also detected in the most severely affected tubules, such as those with necrosis of the tubulocytes (Figure 3F).

### 2.3. Immunohistochemical Nuclear Expression of Histone H2AXY142ph in Normal SHR Kidneys and during AKI

Epigenetic histone modifications with regard to the presence of histone H2AXY142ph were also detected both in sham-operated SHRs (control group) and in rats with induced AKI. However, the extent of expression and some distribution patterns differed among these two groups.

**Figure 4:**
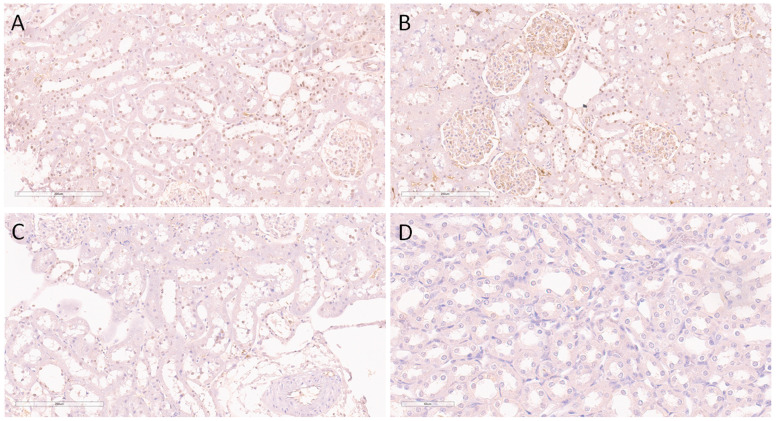
Immunohistochemical nuclear expression of histone H2AXY142ph in sham-operated SHRs. (**A**) Expression in subcapsular kidney cortex involving the majority of nuclei of distal tubules and collecting ducts, as well some nuclei of proximal tubulocytes; (**B**) expression in middle cortical zone detected in the same structures but with slightly decreased abundance; (**C**) juxtamedullary cortical zone with nuclear positivity in less than 25% of tubulocytes; (**D**) absence of expression in the kidney medulla. Magnification ×200 (**A**–**C**), ×400 (**D**).

Figure 4 illustrates the expression of H2AXY142ph in the sham group. It was noticed that this histone was expressed predominantly in the nuclei of distal tubules and collecting ducts, as well in some nuclei of proximal tubular epithelial cells but with a lower intensity.

A varying extent of expression was also detected throughout the cortical kidney parenchyma. Thus, expression was mostly detected within the subcapsular kidney cortical area, involving the majority of the nuclei of distal tubules and collecting ducts, as well some nuclei of the proximal tubulocytes (Figure 4A). In the middle cortical zone, expression was also detected in the same structures but with slightly decreased abundance and in heterogeneous manner, involving up to 50% of the aforementioned structures (Figure 4B). Furthermore, the juxtamedullary cortical zone was also positive for H2AXY142ph but mostly involved less than 25% of the tubulocytes in the zone (Figure 4C). However, despite the presence of collecting ducts in the medulla, this kidney zone was completely negative with regard to H2AXY142ph expression (Figure 4D).

**Figure 5:**
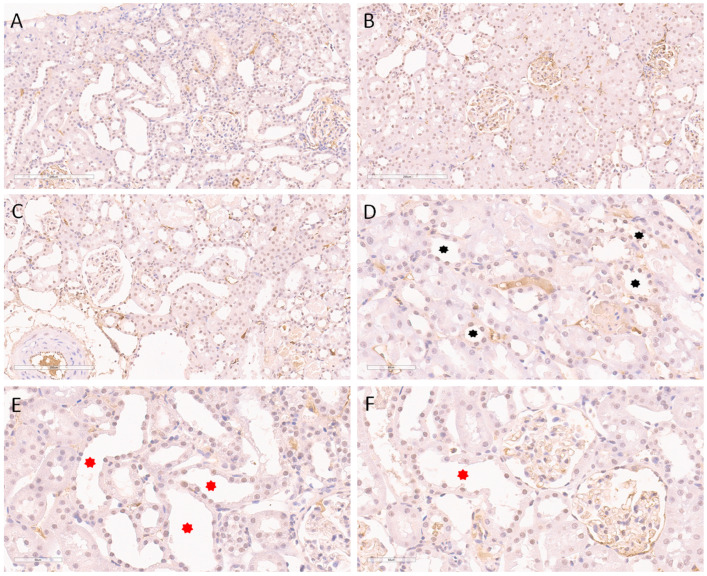
Immunohistochemical nuclear expression of histone H2AXY142ph in animal AKI model. (**A**) Diffuse expression in subcapsular kidney cortex involving the majority of nuclei of proximal tubulocytes; (**B**) abundant expression in middle cortical zone detected in the same structure; (**C**) juxtamedullary cortical zone with nuclear positivity in up to 50% of tubulocytes; (**D**) black asterisks indicate the nuclear expression of histone H2AXY142ph in some tubulocytes within the medulla; (**E**) and (**F**) expression in dilatated proximal tubules, with flattened epithelium (red asterisks), as an early morphological sign of AKI. Magnification ×200 (**A**–**C**), ×400 (**D**–**F**).

A significantly different expression of this type of epigenetically modified H2AX histone was detected in the animal model of AKI, as illustrated in Figure 5, which shows the expression pattern through the cortical region and in the kidney medulla. Compared to the sham group, the expression was more pronounced in the juxtamedullary cortical region (Figure 5C), with a gradual disappearance through the middle cortex (Figure 5B), and the least expression in the subcapsular cortex (Figure 5A). Furthermore, the kidney medulla of the AKI group showed focal expression of H2AXY142ph in the nuclei of some collecting ducts and loops of Henle (Figure 5D). Although the expression of H2AXY142ph was observed only in a minority of normal proximal tubulocytes, mainly in the subcapsular region, in the sham animal group (Figure 4A), experimentally induced AKI provoked the pronounced expression of H2AXY142ph in dilated proximal tubules with a flattened epithelium, as a morphological sign of AKI (Figure 5E,F).

Appreciating the different and highly heterogeneous expression of H2AXY142ph in SHRs with and without AKI induction, we also stained sham-operated, normotensive Wistar rat kidneys for further clarification of the distribution of H2AXY142ph in the kidney parenchyma. In this case, we could not detect any positive nuclei, as shown in Figure S2.

**Figure 6:**
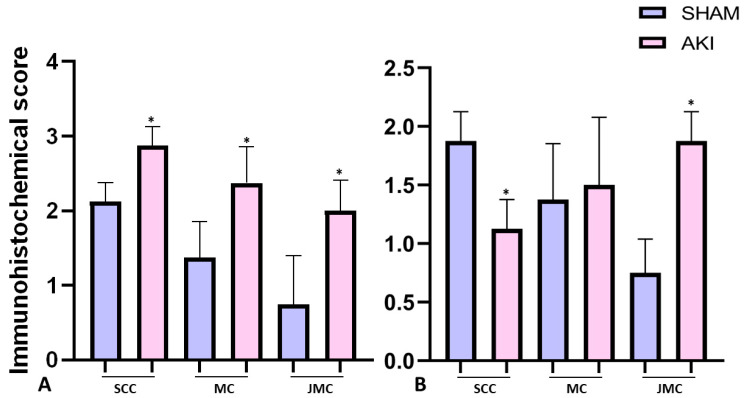
Immunohistochemical expression score for γH2AX(S139) (**A**) and H2AXY142ph (**B**) in the sham-operated SHR and AKI group. Student’s *t*-test for independent samples, * *p* < 0.05, SCC—subcapsular cortical zone, MC—middle cortical zone, JMC—juxtamedullary cortical zone.

We also performed a semiquantitative analysis of the immunohistochemical nuclear expression of histone γH2AX(S139) and H2AXY142ph in normal SHR kidneys and during AKI (Figure 6).

### 2.4. Immunohistochemical Nuclear Expression of Histone H2AX in Normal SHR Kidneys and during AKI

**Figure 7:**
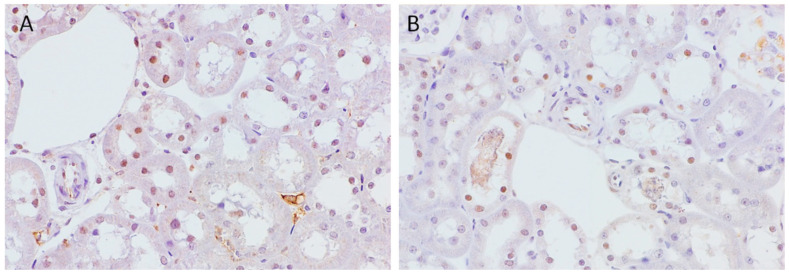
Immunohistochemical nuclear expression of histone H2AX in sham-operated SHRs and in animal AKI model. (**A**) Expression in sham-operated SHRs; (**B**) expression in animal AKI model. Magnification ×400.

Natural, nonepigenetically modified histone H2AX was detected both in sham-operated SHRs (control group) and in rats with induced AKI, but the extent of expression was significantly lower compared to that of epigenetically modified variants in both groups of investigated animals. Thus, in the sham-operated animals, H2AX was detected in the nuclei of all nephron segments but mainly in the subcapsular zone (Figure 7A). On the other hand, AKI animals had lower expression in the tubular structures, and these nephron segments were mainly located in the juxtamedullary zone close to the segments showing necrosis of the tubulocytes (Figure 7B).

In sham-operated, normotensive Wistar rats also, H2AX was detected in the nuclei of all nephron segments but with a higher extent of expression in comparison to the sham-operated SHRs (Figure S3A).

### 2.5. Immunohistochemical Nuclear Expression of Histone H2AZ in Normal SHR Kidneys and during AKI

**Figure 8:**
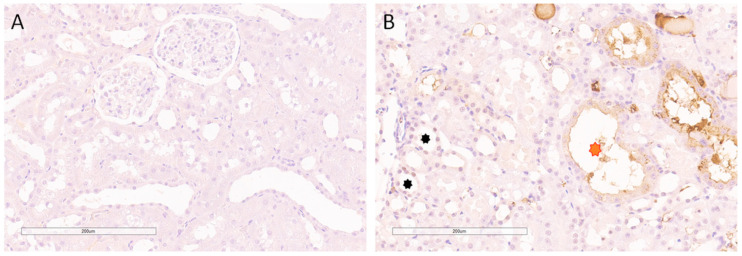
Immunohistochemical nuclear expression of histone H2AZ in sham-operated SHRs and in animal AKI model. (**A**) Absence of expression in sham-operated SHRs; (**B**) scant expression in animal AKI model. Red asterisk indicates dilated tubule with no nuclear expression of histone H2AZ. Black asterisks represent the morphologically preserved tubules close to segments showing necrosis of the tubulocytes. Magnification ×200.

Histone H2AZ was not observed in the control SHR group (Figure 8A), while its expression was noticed in some cell populations in the AKI group, mainly located in the juxtamedullary zone close to the segments showing necrosis of the tubulocytes (Figure 8B).

Normotensive, sham-operated Wistar rats, similarly to sham-operated SHRs, did not express H2AZ (Figure S3B).

### 2.6. Negative Controls for Immunohistochemical Analyses in Investigated Animal Groups

Figure S4 illustrates negative control slides with the complete absence of nuclear staining.

## 3. Discussion

According to our knowledge, this is the first study that evaluates the immunohistochemical patterns of histone H2AX variants’ expression in an experimental model of ischemia–reperfusion-induced acute kidney injury. After the induction of postischemic AKI in spontaneously hypertensive rats, the estimated glomerular filtration rate (eGFR) significantly decreased and was accompanied by extensive renal histopathological changes, confirming that the model was properly conducted. The obtained results were comparable with the decreased single-nephron glomerular filtration rate (snGFR) reported in ischemia–reperfusion injury induced in Munich Wistar rats and detected by linescan multiphoton microscopy [28], as well as by micropuncture studies [29]. Morphological changes in the mentioned studies observed using a fluorescence imaging method [28] and PAS (periodic acid–Schiff)-positive staining [29] were also very similar to the histological findings we confirmed, including variable tubular necrosis accompanied by loss of the proximal tubular morphology with a large number of intracellular casts and cellular debris. In the renal tissue of these rats, we observed a high, diffuse, nuclear expression of phosphorylated H2AX on serine 139 (γH2AX) in the proximal tubules, with decreasing extent from the subcapsular to the medullar region. In the renal medulla we did not detect γH2AX. These findings might be explained by some already published results. Ischemic AKI is a kind of genotoxic stress for the cell, followed by cellular DNA damage [9]. Fernandes et al. and Rogakou et al. showed that, upon DSB induction in mammals, the histone H2AX becomes rapidly phosphorylated at serine 139, serving as a sensitive indicator of DNA DSB formation [15,16]. Similar to these data, Sedelnikova and Bonner, in their study, concluded that γH2AX detection provides a very sensitive, efficient and reproducible measurement of the amount of DNA damage, especially when compared to some other techniques [30]. When damage occurs, the cell can respond in several ways, including DNA repair, checkpoint activity and the initiation of apoptotic pathways [31]. This is known as DNA damage response (DDR). On the other hand, cellular responses during ischemic AKI lead to both lethal and sublethal injuries [8]. In fact, the sensitivity of renal cells to injury depends on different physiological factors including the different positions of the cells belonging to specific parts of the nephron, the ratio of energetic substrate and metabolic demand, oxygen supply, the degree of the postischemic reperfusion and membrane permeability [8]. As proximal tubules are structures that are the most sensitive to ischemic injury [32,33] because of their high metabolic rate and a strong dependence on oxidative phosphorylation [34] as well as findings that proximal tubule cells within the S1 and S2 segments demonstrate a largely reversible injury [35], it seems logical to find an increased diffuse nuclear γH2AX expression in the proximal tubular cells, which we confirmed with our results. If cell death occurs, it is localized primarily in the S3 segment [35].

Today, it is believed that γH2AX serves as a docking buffer for the accumulation and retention of the components of the DDR [36]. It seems that γH2AX has the main role in DDR because it is capable of inducing signals for both the DNA-damage-sensitive cell cycle checkpoints and the DNA repair proteins [31]. The first step provoked by DSBs is to recognize the DNA damage by the accumulation of different sensor proteins, including MDC1, 53BP1 and the MRN complex (MRE11, Rad50, Nbs1) [31,37]. These signals are transmitted to transducers, mainly kinases. The phosphorylation of H2AX activates some of these transducers of which the ataxia-telangiectasia mutated kinase (ATM) is the most important. After that, the sensor information related to the state of the DNA is transmitted to effector kinases, checkpoint kinases 1 and 2. These kinases further regulate downstream effectors such as p53 [38], cell division cycle 25 (Cdc25) [39], Brca 1 and 2 [40] to stop the cell cycle at specific checkpoints and to prevent cell cycle progression when genomic integrity is compromised. This will ensure that DNA stays intact before DNA replication and cell division start [41,42]. The fact that we did not detect γH2AX expression in proximal tubules that were dilated might be explained by the severe injury of these cells. Actually, ischemic AKI is followed by the necrosis and apoptosis of different renal cells. The relative contribution of apoptosis and necrosis to injury varies depending on the severity of the insult [43]. If the degree of the insult is very strong, this will lead to necrosis, which should not be followed by repair mechanism activation.

In addition to the published data indicating the importance of γH2AX in the DDR to DSBs, it was reported that H2AX γ phosphorylation also occurred during apoptosis [44]. However, it was demonstrated that the γH2AX staining pattern in apoptosis was different from the focal distribution observed in DDR [45,46]. After the induction of DDR by different genotoxic factors, γH2AX foci appear, and only thereafter do cells initiate apoptosis and form apoptotic rings. However, when apoptosis is primarily induced by pro-apoptotic agents that do not damage the DNA, the pattern of γH2AX immunostaining directly appears in the form of the apoptotic ring [47]. In the present study, we demonstrated focal nuclear γH2AX immunohistochemical expression, indicating DNA damage rather than primary apoptosis. However, from our immunohistochemical results, we cannot claim that the DNA damage will be repaired, and in case the repair does not occur, thereafter, the cell may go into apoptosis. It was shown that γH2AX appears a few minutes after a DNA lesion. Maximal levels are reached after 30 min, and then, there is a decline with expected disappearance after approximately 24 h [48,49]. According to Hamaski et al. 2007, persistence of the γH2AX foci after the initial induction of DNA damage indicates that some of the damage remains unrepaired [50], helping to identify the cell lines with defective DNA repair [51,52]. As we sacrificed animals 24 h after reperfusion, it seems that most of these cells with nuclear expression of γH2AX were cells that could not be repaired.

In the sham group, the immunohistochemical pattern of γH2AX expression is similar to that in AKI but with a significantly lower expression intensity compared with SHRs with induced postischemic AKI. In addition to increases in the γH2AX levels accompanying the DNA damage induced by genotoxic stress, cells from aging organisms as well as senescing cells in culture display an increased γH2AX signal in the absence of any intentional damage. It was confirmed that γH2AX foci amass in senescing human and primate cell cultures and also in aging mouse tissues including the liver, testes, kidney and lung [53,54,55,56,57,58]. This might potentially be one of the possible explanations for the low expression of γH2AX foci in the sham group also. From the other point of view, we used SHRs in this study, and hypertension is a pathophysiological condition characterized by increased oxidative stress, enhanced vascular inflammation, blood vessel remodeling and increased vascular tone [59]. These factors, especially increased oxidative stress, can be related to genome instability. Zha et al., 2008, reported that γH2AX might be required for the repair of ROS-induced DNA damage [60].

Until now, we still do not know the exact mechanism that determines whether the cell will go into repair or into apoptosis. Cook et al. provided data that, in response to genotoxic stress, a protein tyrosine phosphatase, Eya, encouraged efficient DNA repair rather than apoptosis [23]. They also reported that, in response to DNA damage, Eya specifically dephosphorylated an H2AX C-terminal tyrosine phosphate (H2AXY142ph). Additionally, Banerjee et al. proposed that the persistence of H2AXY142ph phosphorylation after DNA damage leads to apoptosis, while dephosphorylation stimulates repair [61]. In addition to this, Xiao et al. described a qualitative correlation between Y-142 dephosphorylation of H2AX and serine-139 phosphorylation (γH2AX) following DSBs [22]. These findings are in accordance with our results. We showed, in AKI, mild-to-moderate diffuse nuclear immunohistochemical H2AXY142ph expression in the subcapsular region, even in dilated proximal tubule cells, where γH2AX immunohistochemical expression was negative. H2AXY142ph was expressed in distal tubule cells and collecting ducts that also expressed γH2AX. On the other hand, in the medulla, we detected focal nuclear H2AXY142ph expression in the loop of Henle and the collecting ducts even in tubules that were not dilated. From the other point of view, in the sham group, H2AXY142ph was expressed in the same structure as in AKI, but it was distributed with a larger extent than it was in AKI. This time, the extent gradient decreased from the subcapsular region to the medulla, which was opposite to its expression in AKI. Xiao et al. reported that H2AXY142ph is constitutively expressed in cells, by which we can explain the H2AXY142ph expression in the sham group.

In addition to these results, the nuclear expression of the H2AX histone variant, which is a basic component of the nucleosome, was detected in both the sham and AKI groups, and the immunohistochemical pattern of its expression was the inverse of its phosphorylated γH2AX and H2AXY142ph forms’ expression.

When comparing the immunohistochemical nuclear expression of γH2AX and H2AXY142ph in AKI, we can summarize that there is an inverse ratio of these two histone H2AX variants. If we follow different regions from the subcapsular structures to the medulla, there is increasing extent gradient in the nuclear expression of H2AXY142ph, accompanied by a decreasing nuclear expression of γH2AX. Furthermore, we observed that different structures dominated when γH2AX and H2AXY142ph expression levels were compared. γH2AX was expressed only in the proximal tubule, with the exception of when they were dilated. In the medulla, H2AXY142ph was predominantly expressed in the loop of Henle and the collecting ducts.

Distal tubule segments, including the medullary thick ascending limb, are not as sensitive to hypoxia as the proximal tubules because they are more glycolytic [62]. This is one of the reasons why they are relatively resistant to necrosis and why they favor the development of apoptosis [63,64] or sublethal injury. Therefore, we can assume that expressed H2AXY142ph accompanied by absent γH2AX nuclear expression in the distal segments might be the sign of sublethal injury that was successfully repaired. Of course, this should be confirmed in some further studies.

There is a strong association between AKI and the development of CKD [65]. Worldwide, it was reported that 20% of patients with an acute kidney injury episode will develop chronic kidney injury [66]. We still do not know which molecular mechanisms are responsible for the increased risk of chronic kidney disease because of AKI episodes, but there is evidence that epigenetic changes may play a role in this transition from AKI to CKD [65]. It was shown that histone deacetylation stimulates myofibroblast proliferation and epithelial-to-mesenchymal transition [65]. Moreover, it was presented that, in mice with induced unilateral I/R, two histone alterations occur: histone 3, lysine 4 trimethylation (H3K4m3) and increased expression of histone 2 variant (H2AZ). Both changes promote an increase in the expression of fibrotic and inflammatory genes such as MCP-1, TGF-β1 and collagen III [67]. Our results showed moderate sporadic nuclear H2AZ expression mainly in the cells of distal tubules and collecting ducts that were surrounded by dilated tubules with PAS-positive casts. H2AZ expression was absent in the sham group. According to these findings, we can suppose that, very early during AKI, some mechanisms contributing to fibrosis can be activated.

As we used sham-operated SHRs as the control, we also assessed the characterization of histone H2A variants in sham-operated, normotensive Wistar rats. The obtained results were very similar to the immunohistochemical expression pattern in sham-operated SHRs. The only difference involved the absence of H2AXY142ph nuclear expression in sham-operated Wistar rats accompanied by a higher extent of nuclear expression of native H2AX, which may be a possible explanation for the absence of H2AXY142ph, but we need further studies to confirm this speculation.

The limitations of this study include the fact that we performed only immunohistochemical analysis here, but the obtained results are strongly supported by data from the already published literature. We also enhanced the immunohistochemical results with semiquantitative analysis. According to our knowledge, this is the first study that evaluates the immunohistochemical expression of H2AX variants in an experimental model of induced postischemic acute kidney injury, which could be the advantage of this study.

## 4. Materials and Methods

### 4.1. Ethics Statement

The experimental protocol was approved by the Ethics Committee of the Institute for Medical Research, University of Belgrade, Serbia (No. 323-0702569/2018-05/2), according to the National Law on Animal Welfare (“Službeni Glasnik” No. 41/09), which is consistent with the guidelines for animal research and principles of the European Convention for the Protection of Vertebrate Animals Used for Experimental and Other Purposes (Official Daily N. L 358/1-358/6, 18, December 1986) and the Directive on the protection of animals used for scientific purposes (Directive 2010/63/EU of the European Parliament and of the Council, 22 September 2010).

### 4.2. Animals

Male, spontaneously hypertensive rats (SHRs, descendants of breeders originally obtained through Taconic Farms, Germantown, NY, USA) and normotensive Wistar rats, 24 weeks old and about 300 g in weight were used in this study. The animals were bred at the Institute for Medical Research, University of Belgrade, Serbia, and kept under controlled laboratory conditions (constant temperature 22 ± 1 °C, humidity of 65 ± 1%, 12 h light/dark cycle). The animals were housed in groups of four rats per cage and fed a standard chow for laboratory rats (Veterinarski zavod, Subotica, Serbia) with free access to food and water.

### 4.3. Experimental Design

Hypertension was confirmed in all rats by indirect measurement on the tail artery (Narco Bio Systems INC, Houston, TX, USA). The animals were randomly divided into three experimental groups: sham-operated, normotensive Wistar rats (W-SHAM, *n* = 4); sham-operated SHRs (SHR-SHAM, *n* = 4) and SHRs with induced postischemic AKI (AKI, *n* = 4).

All surgical procedures were performed in anesthetized rats by injecting 35 mg/kg b.m. sodium pentobarbital intraperitoneally. AKI was induced by the removal of the right kidney and atraumatic clamp occlusion of the left renal artery for 45 min. In the sham-operated group, an identical surgical procedure was performed but without left renal artery clamping. At the end of the AKI procedure, the abdominal incision wound was surgically closed, and the rats were placed into cages for 24 h, with free access to food and water.

### 4.4. Estimated Glomerular Filtaration Rate (eGFR)

Blood samples, obtained by puncture of the abdominal aorta, were collected into tubes containing lithium heparin (Li-heparin, Sigma-Aldrich, St. Louis, MO, USA) and used for further analysis. Blood was centrifuged to separate the plasma. Until assaying, the plasma samples were stored at −20 °C. Plasma creatinine and urea were measured using the automatic COBAS INTEGRA 400 plus (Hoffmann-La Roche, Munich, Germany) analyzer.

In order to calculate the eGFR, the following formulas [68] were used:

eGFR = 880 × W^0.695^ × C^−0.660^ × U^−0.391^ (if plasma creatinine < 52 µmol/L)

eGFR = 5862 × W^0.695^ × C^−1.150^ × U^−0.391^ (if plasma creatinine ≥ 52 µmol/L), (1)

where eGFR is the estimated GFR (µL/min), W is the weight (g), C is the plasma creatinine concentration (µmol/L), and U is the plasma urea concentration (mmol/L).

### 4.5. Immunochistochemical Analysis

Immunohistochemistry was applied on formalin-fixed, paraffin-embedded kidney samples. Four-micrometer-thick paraffin sections were subjected to deparaffinization and hydration steps and afterward introduced to heat-induced antigen retrieval in a citrate buffer (pH 6.0). Novolink™ Polymer Detection System components (Leica Biosystems, Wetzlar, Germany) were applied according to the manufacturer’s instructions for the immunohistochemistry protocol. A peroxidase block (5 min. incubation time) and protein blocks (5 min.) were applied prior to incubation with primary antibody for 1 h at room temperature. The following primary antibodies (Abcam, Cambridge, UK) were used: anti-H2A.X (ab11175, 1:1000), anti-gamma H2A.X (phospho S139) (ab11174, 1:1000), anti-H2A.X (phospho Y142) (ab94602, 5 µg/mL), anti-H2A.Z (ab11175, 1:1000). Secondary antibodies were applied from the Novolink™ Polymer Detection System Kit (Leica Biosystems, Wetzlar, Germany). This kit supplied “ready to use” postprimary antibody and, according to its manufactural protocol, was incubated for 30 min. at room temperature, followed by the application of Novolink™ Polymer for 30 min. at room temperature. Visualization of the antigen–antibody reaction was achieved by applying 3,3′-diaminobenzidine (DAB) for 5 min. (brown products). Subsequent counterstaining with hematoxylin (30 s) was conducted. Negative controls were created for all samples by omitting the primary antibody and were instead incubated for the same time with phosphate-buffered saline (PBS) only. All other steps during the immunostaining procedure were the same as performed in the experimental groups. Slides were evaluated using the light microscope BX53 with a DP70 camera (Olympus, Hamburg, Germany). The evaluation was performed by two independent pathologists, blinded to the experimental information.

For immunohistochemical scoring, according to the extent of expression in different kidney structures, the following parameters were semiquantitatively evaluated: 0—without expression, 1—up to 25% positive nuclei, 2—from 25 to 50% positive nuclei, 3—more than 50% positive nuclei. The sum of these changes represented the immunohistochemical scores for each parameter, and they were used for comparison between groups.

### 4.6. Statistical Analysis

All data are expressed as the mean ± standard deviation (SD). A statistical analysis of each of the parameters of interest was carried out using Student’s *t*-test for independent samples and analysis of variance (one-way ANOVA). When a significant F-value was obtained in the one-way ANOVA test (*p* < 0.05), a post hoc test (Tukey’s HSD multiple comparisons test) was used. A *p*-value <0.05 was considered significant. The statistical calculations were performed using GraphPad Prism for Windows (Version 7.0, GraphPad Software, La Jolla, CA, USA).

## 5. Conclusions

In conclusion, we can point out that postischemic AKI induced in SHRs is followed by a strong, diffuse nuclear γH2AX expression in the proximal tubule, with a decreasing extent gradient from the subcapsular region to the medulla. On the contrary, H2AXY142ph, was expressed in the loop of Henle, distal tubules and collecting ducts, as well as in proximal tubules with signs of acute kidney injury, such as tubular dilatation. These findings may indicate the degree of DNA damage, followed by postischemic AKI, with potential clinical and prognostic implications regarding this condition.

## Data Availability

The data presented in this study are available in the article.

## Supplementary Materials

The following are available online at https://www.mdpi.com/article/10.3390/ijms24098085/s1.
