# Supplementary material for: Immunohistochemical Pattern of Histone H2A Variant Expression in an Experimental Model of Ischemia–Reperfusion-Induced Acute Kidney Injury
Source: Int J Mol Sci. 2023 Apr 30;24(9):8085. doi: 10.3390/ijms24098085 (PMC10179385; doi:10.3390/ijms24098085)
Supplement: Supplementary file 1 [file ijms-24-08085-s001.zip › ijms-2334664-supplementary.pdf]

Supplementary Materials:

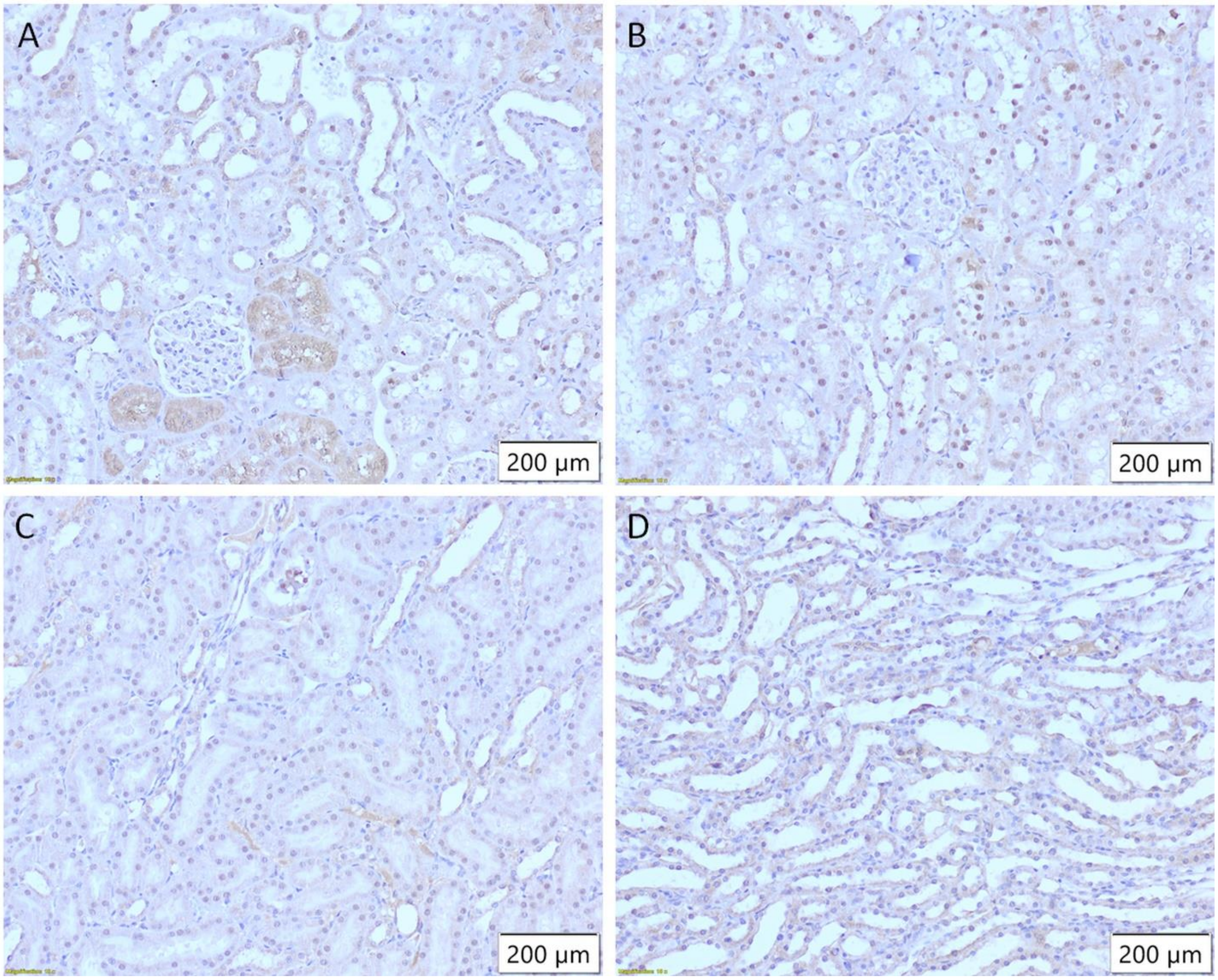

Figure S1: Immunohistochemical nuclear expression of histone γH2AX(S139) in sham-operated Wistar rats. A) expression in subcapsular kidney cortex involving the majority of nuclei of proximal tubulocytes; B) expression in the middle cortical zone detected in the same structures; C) juxtamedullary cortical zone with nuclear positivity in less than 25% of tubulocytes; D) the absence of expression in the kidney medulla.

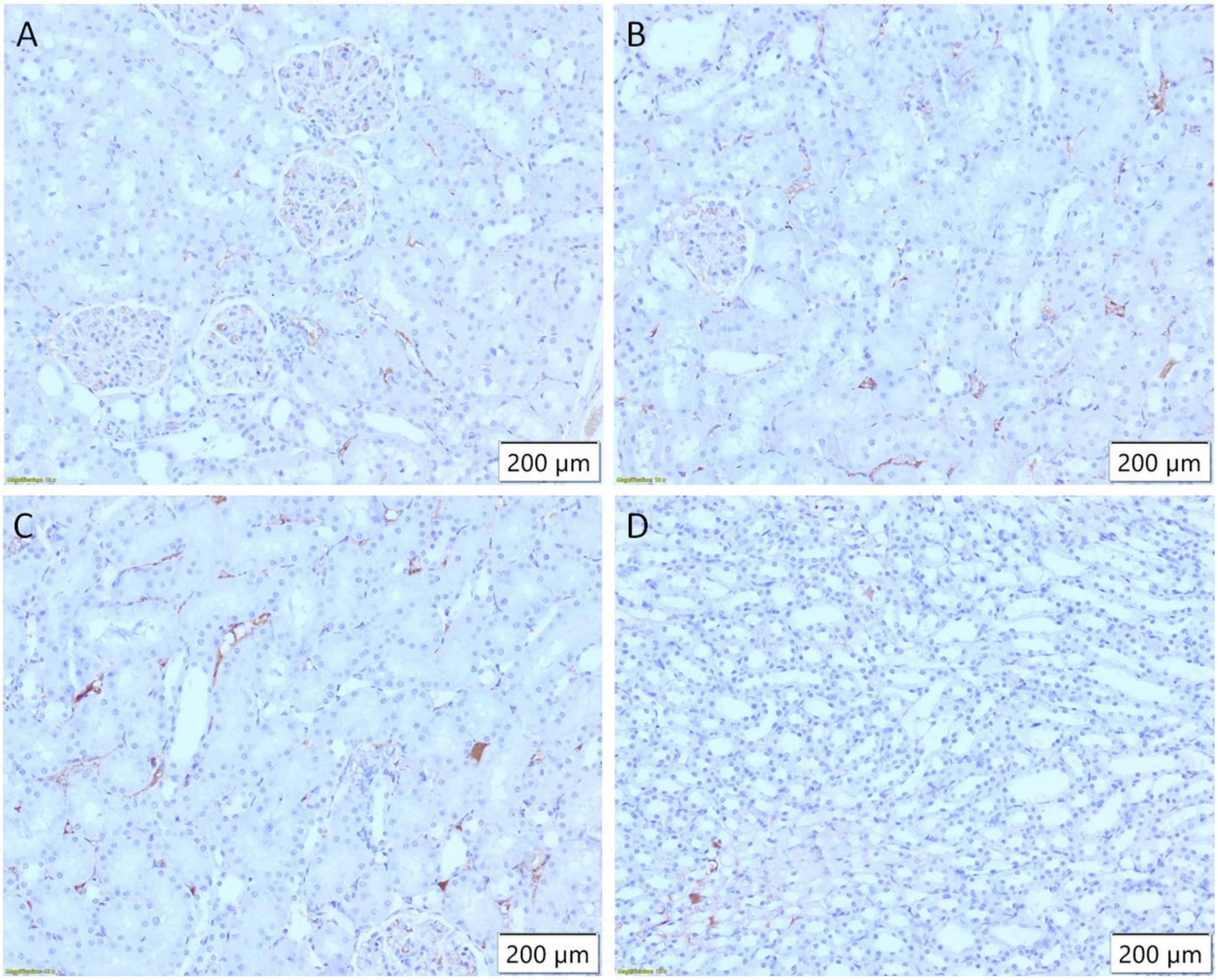

Figure S2: The absence of immunohistochemical nuclear expression of histone H2AXY142ph in sham-operated Wistar rats. A) subcapsular kidney cortex; B) middle cortical zone; C) juxtamedullary cortical zone; D) kidney medulla.

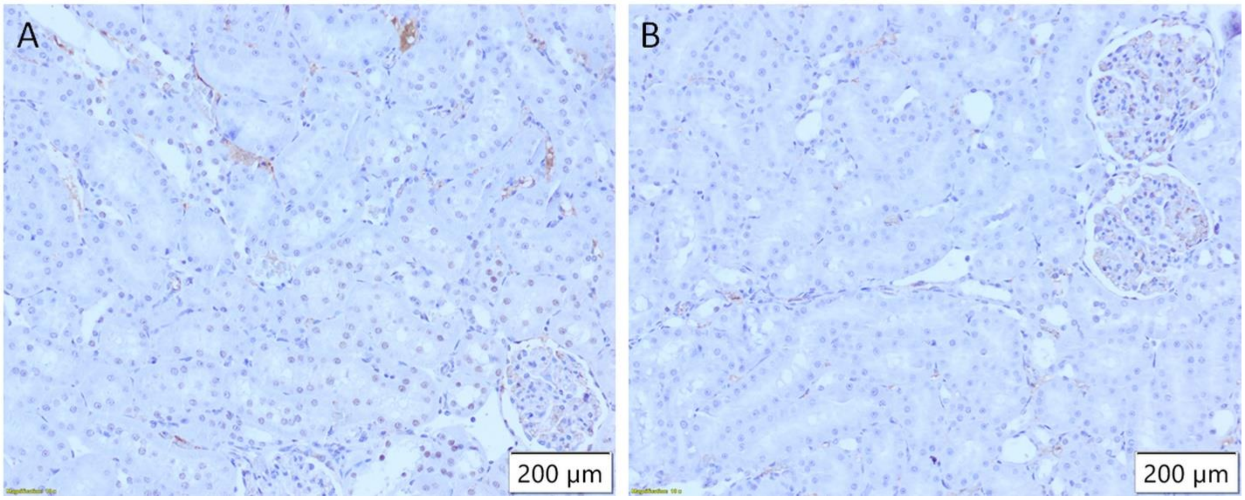

Figure S3: Immunohistochemical detection of histone H2AX and H2AZ in Wistar rats. A) nuclear expression of histone H2AX in some tubulocytes; B) the absence of expression of H2AZ histone.

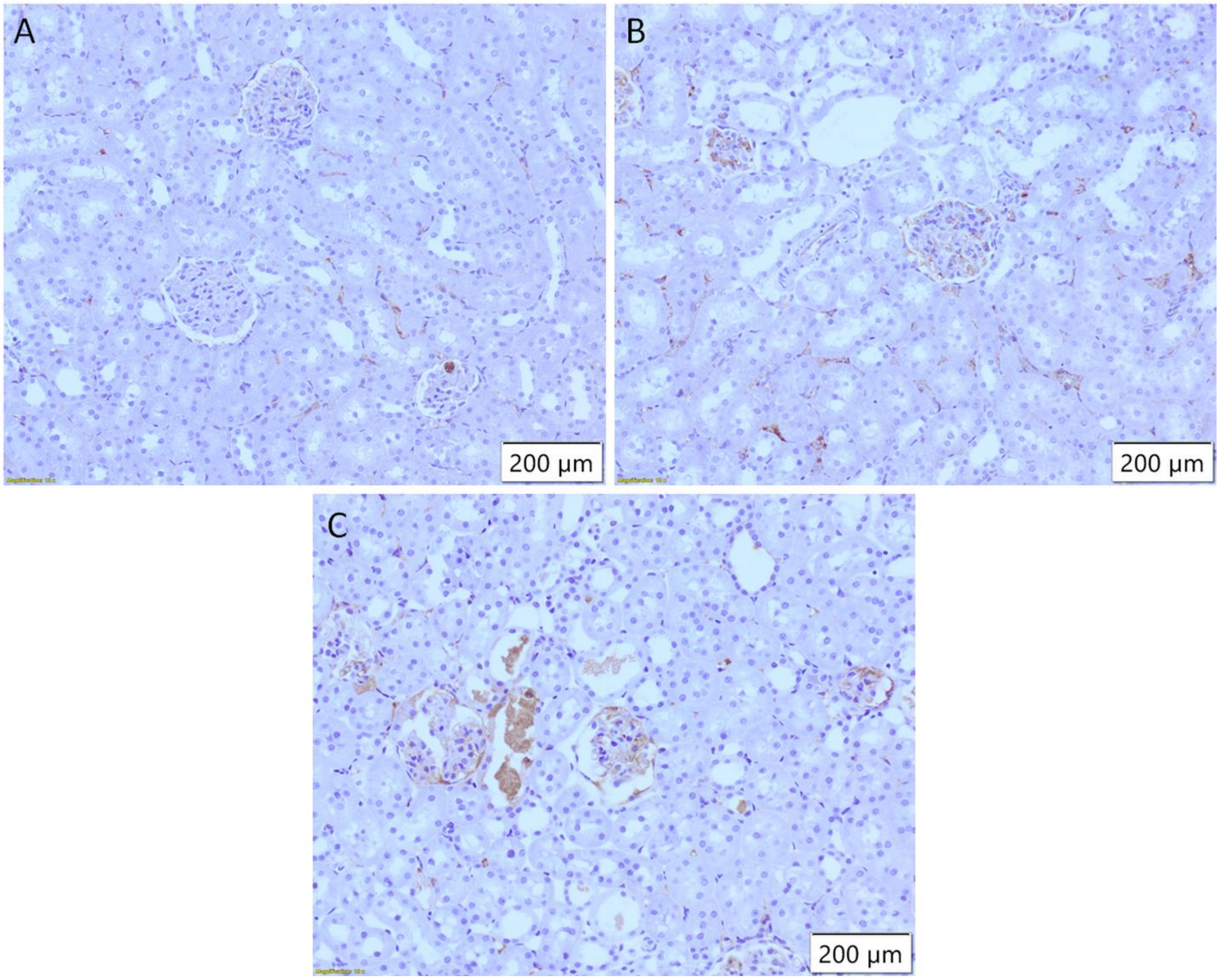

Figure S4: Negative controls for immunohistochemical analyses in investigated animal groups. A) Wistar rat sham- operated kidney; B) SHR rat sham-operated kidney; C) SHR rat with induced AKI showed unspecific DAB binding to necrotic material within tubular luminal area, but without any nuclear staining.
